# Characteristic columnar connectivity caters to cortical computation: Replication, simulation, and evaluation of a microcircuit model

**DOI:** 10.3389/fnint.2022.923468

**Published:** 2022-10-03

**Authors:** Tobias Schulte to Brinke, Renato Duarte, Abigail Morrison

**Affiliations:** ^1^Institute of Neuroscience and Medicine (INM-6) and Institute for Advanced Simulation (IAS-6) and JARA-BRAIN Institute I, Jülich Research Centre, Jülich, Germany; ^2^Department of Computer Science 3 - Software Engineering, RWTH Aachen University, Aachen, Germany; ^3^Donders Institute for Brain, Cognition and Behavior, Radboud University, Nijmegen, Netherlands

**Keywords:** neocortex, cortical column, microcircuit, connectivity structure, neuron dynamics, replication, reproduction

## Abstract

The neocortex, and with it the mammalian brain, achieves a level of computational efficiency like no other existing computational engine. A deeper understanding of its building blocks (cortical microcircuits), and their underlying computational principles is thus of paramount interest. To this end, we need reproducible computational models that can be analyzed, modified, extended and quantitatively compared. In this study, we further that aim by providing a replication of a seminal cortical column model. This model consists of noisy Hodgkin-Huxley neurons connected by dynamic synapses, whose connectivity scheme is based on empirical findings from intracellular recordings. Our analysis confirms the key original finding that the specific, data-based connectivity structure enhances the computational performance compared to a variety of alternatively structured control circuits. For this comparison, we use tasks based on spike patterns and rates that require the systems not only to have simple classification capabilities, but also to retain information over time and to be able to compute nonlinear functions. Going beyond the scope of the original study, we demonstrate that this finding is independent of the complexity of the neuron model, which further strengthens the argument that it is the connectivity which is crucial. Finally, a detailed analysis of the memory capabilities of the circuits reveals a stereotypical memory profile common across all circuit variants. Notably, the circuit with laminar structure does not retain stimulus any longer than any other circuit type. We therefore conclude that the model's computational advantage lies in a sharper representation of the stimuli.

## 1. Introduction

Neurons of the neocortex are arranged in layers, forming connectivity structures through their synapses that share many properties across various brain areas. This suggests that diverse cortical areas are likely based on a common microcircuit template (see e.g., Mountcastle, 1997; Horton and Adams, 2005; DeFelipe, 2012; Harris and Shepherd, 2015). These broad commonalities suggest a functional purpose behind this structure that gives networks an information processing advantage over randomly connected circuits.

To investigate this hypothesis, Stefan Häusler and Wolfgang Maass developed a data-based microcircuit model and tested its computational properties in comparison with networks with equivalent dynamics but alternative connectivity structures in their seminal paper *A Statistical Analysis of Information-Processing Properties of Lamina-Specific Cortical Microcircuit Models* (Häusler and Maass, 2007).

After this paper was published as one of the first studies on data-based cortical column models, it was cited hundreds of times and influenced the computational neuroscience community's view on the purpose and benefits of a laminar cortical network structure. Since then, the model has been used by Wolfgang Maass's team to analyze, for example, the distributions of network motifs in its connectivity structure (Häusler et al., 2009) and to show how a version of the network with stochastic neurons can exploit noise for computation (Habenschuss et al., 2009; Maass, 2014). Rasch et al. (2011) use the model as the basis for a larger network that also includes a model of the retina and lateral geniculate nucleus (LGN) of the thalamus to analyze its responses to natural stimuli and compare them with *in vivo* activity. Since the publication of Häusler and Maass (2007), modeling of cortical columns has partially evolved toward large-scale networks of point neurons whose focus is to accurately reproduce the statistical properties of spike activity from *in vivo* data (e.g., Potjans and Diesmann, 2014). The other recent direction of modeling cortical columns focuses on large networks of biophysically detailed neural compartment models, such as Markram et al. (2015) and Billeh et al. (2020). Nevertheless, smaller network models continue to be relevant because simulating these large-scale models requires huge amounts of computing resources that are beyond the scope of many computational laboratories. Therefore, as a highly influential study uncovering the relationships between structure, dynamics and function, it would be of great benefit to have the computational model introduced by Häusler and Maass (2007) available for further study and quantitative comparison with other models.

Unfortunately, as the model was originally implemented in MATLAB (unknown version, but no later than R2006b) and the C++ simulation plugin *csim* is no longer maintained, the code can no longer be executed. In this article we present a replication of the original study, which serves the twin purpose of testing the original findings and providing an executable version of the model to the computational neuroscience community. Specifically, we re-implement their model using the open source softwares *NEST* (Hahne et al., 2021) to simulate the networks, *NESTML* (Babu et al., 2021) to define the neuron model and Python for data analysis, thus ensuring a reusable and maintainable code base.

Here, we use the term *replication* in the R^5^ sense described by Benureau and Rougier (2018), i.e., striving to obtain the same results using an independent code base, whereas a *reproduction* (R^3^) of the model would have been achieved if we had obtained the results of the original study using the original code. Note that others have argued that these terms should be used the other way around: see Plesser (2018) for an overview and analysis.

Following the structure of the original work, we construct a cortical column model based on data from rat and cat cortical areas published by Thomson (2002). The network consists of spiking Hodgkin-Huxley neurons with an intrinsic conductance-based noise mechanism that represents the incoming currents generated by stochastically releasing synapses and is connected by synapses with short-term plasticity. Using this network model, we investigate the impact of the data-based laminar structure on the computational performance of the system. Besides the data-based model, we implement additional control models that share the global statistics of the microcircuit whilst removing specific network properties. This allows analysis of how different network properties affect the networks' computational performance on various tasks based on input signals that are encoded as precise spike patterns or spike trains with changing firing rates.

These tasks are designed in such a way that they allow us to draw conclusions about computational abilities of the models under investigation by testing the networks not only on their simple classification capabilities, but also on memory and their nonlinear processing power. Following the reservoir computing paradigm, the synaptic efficacies of the recurrent connections within the network are not trained to improve performance; only the projections from the network to separate readout neurons are learned.

We successfully reproduce the main data-based model and all six control circuit variants. The results on the computational tasks confirm the findings of the original study, most notably that the data-based circuit has superior computational performance to circuits without laminar structure.

Going beyond the experiments of the original study, and demonstrating the value of having executable versions of important models, we further examine the generalizability of the results with respect to the neuron model. Assuming that the laminar structure is the most important component of the model, we hypothesize that the central findings are not dependent on the specific choice of the somewhat complex Hodgkin-Huxley neurons used in the original study. To investigate this hypothesis, we simplify the neuron model by reducing its complexity to basic integrate-and-fire dynamics and show that this simplification not only maintains the superior performance of the data-based circuit, but even increases its absolute performance on almost all tasks. The same is true for the removal of the noise mechanism from the Hodgkin-Huxley model. Although noise was added mainly to increase biological plausibility rather than to improve performance, it is not necessarily the case that noise degrades the performance of a neural system, since, for example, effects such as stochastic resonance can improve the detection of weak signals (Wiesenfeld and Moss, 1995; McDonnell and Ward, 2011).

Finally, we extend the original computational tasks to include a more detailed examination of the memory capabilities of the systems under consideration, reflecting the fact that the ability to recall information over time forms the basis for a variety of cognitive processes. Our results reveal a stereotypical memory profile for all tested circuits and demonstrate that the characteristic temporal structure of the stimulus has differential effects on the task performance of the networks receiving it.

Apart from providing a reproducible and re-usable implementation of the cortical microcircuit model in Häusler and Maass (2007), our successful replication reduces the likelihood that the original findings were influenced by implementation errors (Benureau and Rougier, 2018; Pauli et al., 2018). Our findings thus lend further support to the hypothesis that the highly nonrandom connectivity structure of cortical columns serves important computational purposes, with the degree distributions, i.e., the distributions of the number of incoming and outgoing connections per neuron, playing the most prominent role. Going beyond the original findings, we further demonstrate that the computational benefits of the laminar structure are not dependent on the complexity of the neuron model. Finally, we discover that the laminar structure does not confer memory benefits in the model—the circuits with laminar structure do not retain stimulus information for longer than networks with other connectivity assumptions—and conclude that the superior computational performance is achieved primarily by generating more distinct stimulus representations.

## 2. Materials and methods

### 2.1. Microcircuit model

In the following sections we provide details of our implementation of the microcircuit model that is publicly available at Zenodo (Schulte to Brinke et al., 2022) and compare it to the model described in Häusler and Maass (2007), whose implementation is available at ModelDB (McDougal et al., 2017; accession number 82385, https://senselab.med.yale.edu/ModelDB/showmodel?model=82385).

#### 2.1.1. Neuron model

The networks consist of single-compartment Hodgkin-Huxley type neurons with three different active currents, as described by Destexhe and Paré (1999), and an intrinsic conductance noise mechanism introduced by Destexhe et al. (2001):


(1) $$\begin{array}{l} C_{m}\frac{dV_{m}}{dt}=-g_{L}\left(V_{m}-E_{L}\right)-I_{Na}-I_{K}-I_{M}-I_{noise} \end{array}$$

where *V*_*m*_ is the membrane potential, *C*_*m*_ is the membrane capacitance, *g*_*L*_ is the leak conductance and *E*_*L*_ is the leak reversal potential. *I*_*Na*_ is a voltage-dependent Na^+^ current with the following dynamics:


(2) $$\begin{array}{l} I_{Na}=g_{Na}m^{3}h\;\left(V_{m}-E_{Na}\right) \end{array}$$

(3) $$\begin{array}{l} \frac{dm}{dt}=\alpha_{m}\left(V_{m}\right)\left(1-m\right)-\beta_{m}\left(V_{m}\right)m \end{array}$$

(4) $$\begin{array}{l} \frac{dh}{dt}=\alpha_{h}\left(V_{m}\right)\left(1-h\right)-\beta_{h}\left(V_{m}\right)h \end{array}$$

(5) $$\begin{array}{l} \alpha_{m}=\frac{-0.32\left(V_{m}-V_{T}-13\right)}{\text{exp}\left[-\left(V_{m}-V_{T}-13\right)/4\right]-1} \end{array}$$

(6) $$\begin{array}{l} \beta_{m}=\frac{0.28\left(V_{m}-V_{T}-40\right)}{\text{exp}\left[\left(V_{m}-V_{T}-40\right)/5\right]-1} \end{array}$$

(7) $$\begin{array}{l} \alpha_{h}=0.128\text{ exp}\left[-\left(V_{m}-V_{T}-V_{S}-17\right)/18\right] \end{array}$$

(8) $$\begin{array}{l} \beta_{h}=\frac{4}{1+\text{exp}\left[-\left(V_{m}-V_{T}-V_{S}-40\right)/5\right]} \end{array}$$

where *g*_*Na*_ is the sodium peak conductance, *E*_*Na*_ is the sodium reversal potential, *V*_*S*_ is a voltage that shifts the inactivation toward hyperpolarized values and *V*_*T*_ is a voltage offset that controls dynamics and adjusts the membrane threshold. Note, the model does not incorporate an explicit threshold; the membrane potential threshold *V*_*thresh*_ in Table 1 is just the potential at which the peak in the membrane potential is recognized as a spike by the simulator. This is also the reason why a refactory period *t*_*ref*_ is needed to avoid the emission of multiple spikes during a peak in the membrane potential. *I*_*K*_ is a delayed-rectifier K^+^ current:


(9) $$\begin{array}{l} I_{K}=g_{K}n^{4}\left(V_{m}-E_{K}\right) \end{array}$$

(10) $$\begin{array}{l} \frac{dn}{dt}=\alpha_{n}\left(V_{m}\right)\left(1-n\right)-\beta_{n}\left(V_{m}\right)n \end{array}$$

(11) $$\begin{array}{l} \alpha_{n}=\frac{-0.032\left(V_{m}-V_{T}-15\right)}{\text{exp}\left[-\left(V_{m}-V_{T}-15\right)/5\right]-1} \end{array}$$

(12) $$\begin{array}{l} \beta_{n}=0.5\text{ exp}\left[-\left(V_{m}-V_{T}-10\right)/40\right] \end{array}$$

**Table 1:** Neuron parameters.

**Parameter**	**Value**	**Source**	**Description**
*V*_*m*_	uniformly distributed between -70 and –60 mV	Paper	Membrane potential
*V*_*T*_	–63 (–58) mV	Code (Destexhe and Paré, 1999)	Voltage offset that controls dynamics
*V*_*thresh*_	–30 mV	Code	Membrane potential threshold
*V*_*S*_	–10 mV	Destexhe and Paré, 1999	Shifting voltage
*E*_*L*_	–80 mV	Destexhe et al., 2001	Leak reversal potential
*a*	34,636 μm^2^	Paper	Membrane area used for all but *g*_*M*_
ρ_*C*_*m*__	1 μF/cm^2^	Destexhe et al., 2001	Membrane capacitance density
*C*_*m*_	346.36 pF	*a* · ρ_*C*_*m*__	Capacity of the membrane
ρ_*g*_*L*__	0.045 mS/cm^2^	Destexhe et al., 2001	Leak conductance density
*g*_*L*_	15.5862 nS	*a* · ρ_*g*_*L*__	Leak conductance
τ_*sy*_*n*__*ex*__	3 ms	Code	Time constant of the excitatory synaptic exponential function
τ_*sy*_*n*__*in*__	6 ms	Code	Time constant of the inhibitory synaptic exponential function
*t*_*ref*_	3 ms	Code	Duration of refactory period
*E*_*ex*_	0 mV	Destexhe et al., 2001	Excitatory synaptic reversal potential
*E*_*in*_	–75 mV	Destexhe et al., 2001	Inhibitory synaptic reversal potential
*E*_*Na*_	50 (60) mV	Code (Mainen et al., 1995)	Sodium reversal potential
ρ_*g*_*Na*__	516 (500) pS/μm^2^	Code (paper)	Peak conductance density for *I*_*Na*_
*g*_*Na*_	17,872.176 nS		
(17,318 nS)	*a* · ρ_*g*_*Na*__ Code (paper)	Sodium peak conductance	
*E*_*K*_	–90 mV	Code	Potassium reversal potential
ρ_*g*_*K*__	100 pS/μm^2^	Paper	Peak conductance density for *I*_*K*_
*g*_*K*_	3,463.6 nS	*a* · ρ_*g*_*K*__	Potassium peak conductance
*a*_*M*_	10,000 μm^2^	Code	Membrane area used for *g*_*M*_
*E*_*M*_	–80 (–90) mv	Code (Mainen et al., 1995)	Potassium reversal potential for *I*_*M*_
ρ_*g*_*M*__	10 (5) pS/μm^2^	Code (paper)	Peak conductance density for *I*_*M*_
*g*_*M*_*ex*__	100 (173.18) nS	*a*_*M*_ · ρ_*g*_*M*__ Code (*a* · ρ_*g*_*M*__paper)	Peak conductance of additional potassium ion channel in exc. neurons
*g*_*M*_*in*__	0 nS	Paper	Peak conductance of additional Potassium ion channel in inh. neurons

The source column indicates where the value can be found, searching in the following order: main replicated paper, referenced papers, source code. If a value was given in the paper which differs from the one used in the code, the paper value is written in parenthesis.

Here, *g*_*K*_ is the potassium peak conductance and *E*_*K*_ is the potassium reversal potential. The third current is a non-inactivating K^+^ current responsible for spike frequency adaptation, which is only activated for excitatory neurons and was first described by Mainen et al. (1995):


(13) $$\begin{array}{l} I_{M}=g_{M}p\left(V_{m}-E_{M}\right) \end{array}$$

(14) $$\begin{array}{l} \frac{dp}{dt}=\alpha_{p}\left(V_{m}\right)\left(1-p\right)-\beta_{p}\left(V_{m}\right)p \end{array}$$

(15) $$\begin{array}{l} \alpha_{p}=\frac{r\cdot \left(V_{m}+30\right)}{1-\text{exp}\left[-\left(V_{m}+30\right)/9\right]} \end{array}$$

(16) $$\begin{array}{l} \beta_{p}=\frac{-r\cdot \left(V_{m}+30\right)}{1-\text{exp}\left[-\left(V_{m}+30\right)/9\right]} \end{array}$$

where *g*_*M*_ is the peak conductance for this additional potassium channel. The factor *r* is set to 0.0001 by Destexhe et al. (2001), but Häusler and Maass (2007) use a value of 0.001 in their code, and we followed the latter in our implementation. A complete specification of all neuron parameters can be found in Table 1.

In addition to these ion channel dynamics, Häusler and Maass (2007) used noisy background currents whose conductances are modeled by an Ornstein-Uhlenbeck process. This stochastic background activity was introduced by Destexhe et al. (2001) to represent the spontaneous activations of incoming synapses as follows:


(17) $$\begin{array}{l} I_{noise}=g_{ne}\left(t\right)\left(V_{m}-E_{ex}\right)+g_{ni}\left(t\right)\left(V_{m}-E_{in}\right) \end{array}$$

where *g*_*ne*_ is the time dependent excitatory conductance, *E*_*ex*_ is the excitatory synaptic reversal potential and *g*_*ni*_ and *E*_*in*_ are their inhibitory counterparts.

The conductances are calculated by the following update rules:


(18) $$\begin{array}{l} g_{ne}\left(t+h\right)=g_{e0}+\left[g_{ne}\left(t\right)-g_{e0}\text{ exp}\left(-h/\tau_{ne}\right)+A_{e}N_{1}\left(0,1\right)\right] \end{array}$$

(19) $$\begin{array}{l} g_{ni}\left(t+h\right)=g_{i0}+\left[g_{ni}\left(t\right)-g_{i0}\text{ exp}\left(-h/\tau_{ni}\right)+A_{i}N_{2}\left(0,1\right)\right] \end{array}$$

where *g*_*e*0_ and *g*_*i*0_ are average conductances, τ_*ne*_ and τ_*ni*_ are time constants, *h* is the integration step, *N*_1_(0, 1) and *N*_2_(0, 1) are random numbers drawn from a normal distribution with mean 0 and standard deviation 1, and *A*_*e*_ and *A*_*i*_ are amplitude coefficients given by


(20) $$\begin{array}{l} A_{e}=\sqrt{\frac{D_{e}\tau_{ne}}{2}\left[1-\text{exp}\left(\frac{-2h}{\tau_{ne}}\right)\right]} \end{array}$$

(21) $$\begin{array}{l} A_{i}=\sqrt{\frac{D_{i}\tau_{ni}}{2}\left[1-\text{exp}\left(\frac{-2h}{\tau_{ni}}\right)\right]} \end{array}$$

*D*_*e*_ and *D*_*i*_ are noise diffusion coefficients:


(22) $$\begin{array}{l} D_{e}=\frac{2\sigma_{ne}^{2}}{\tau_{ne}} \end{array}$$

(23) $$\begin{array}{l} D_{i}=\frac{2\sigma_{ni}^{2}}{\tau_{ni}} \end{array}$$

where σ_*ne*_ and σ_*ni*_ are standard deviations of the excitatory and inhibitory noise conductances, respectively. All of the parameter values for the noise term can be found in Table 2. The neuron model also handles synaptic conductances, which increase immediately with each spike and then decay exponentially with time constants τ_*sy*_*n*__*ex*__ for spikes coming from excitatory neurons and τ_*sy*_*n*__*in*__ for inhibitory ones. To implement the full neuron model we described it in NESTML (Babu et al., 2021), from which code can be automatically generated for NEST 3.0 (Hahne et al., 2021).

**Table 2:** Neuronal conductance noise parameters; source definition as in Table 1.

**Parameter**	**Value**	**Source**	**Description**
τ_*ne*_	2.7 ms	Paper	Time constant for the excitatory noise conductance
τ_*ni*_	10.5 ms	Paper	Time constant for the inhibitory noise conductance
*g*_*ne*_	12 nS	Paper	Mean conductance of the excitatory noise
*g*_*ni*_	57 nS	Paper	Mean conductance of the inhibitory noise
σ_*ne*_	3 nS	Paper	Standard deviation of the excitatory noise conductance
σ_*ni*_	6.6 nS	Paper	Standard deviation of the inhibitory noise conductance

#### 2.1.2. Neuron model variations

We examine the robustness of the model results to simplification of the neuron model described above. The first variation we apply is to disable the intrinsic conductance noise mechanism by setting σ_*ne*_ and σ_*ni*_ to 0. This adjustment also allows us to study the susceptibility of the networks to noise in the system. In a second step, we additionally disable the *I*_*Na*_, *I*_*K*_, and *I*_*M*_ currents, resulting in leaky integrate-and-fire (iaf) neurons. We leave all parameters unrelated to these ion channel currents unchanged, but change the membrane potential threshold *V*_*thresh*_ of each population (L2/3-E: −52 *mV*, L2/3-I: −55 *mV*, L4-E: −49 *mV*, L4-I: −55 *mV*, L5-E:−57.0 *mV*, L5-I: −65.0 *mV*) such that the means of the population firing rates of the data-based circuit with integrate-and-fire neurons match those of the network with Hodgkin-Huxley neurons as closely as possible (see Supplementary materials for firing rate distributions of all networks).

#### 2.1.3. Synapse model

For the synaptic short-term dynamics, we use the tsodyks2_synapse model implemented in NEST. This model implements short-term synaptic plasticity according to Maass and Markram (2002) with the following equations, which are also used in the replicated paper:


(24) $$\begin{array}{l} A_{k}=w\cdot u_{k}\cdot R_{k} \end{array}$$

where *A*_*k*_ is the amplitude of the postsynaptic potential for the *k*th spike and *w* is the synaptic weight. The release probability *u*_*k*_ is given by:


(25) $$\begin{array}{l} u_{k}=U+u_{k-1}\left(1-U\right)\text{ exp}\left(-\frac{\Delta_{k-1}}{\tau_{fac}}\right) \end{array}$$

where *U* determines the increase in *u* with each spike, Δ_*k*_ denotes the time since the last spike and τ_*fac*_ is the time constant for recovery from facilitation. *R*_*k*_ is the fraction of synaptic efficacy available for the *k*th spike and follows:


(26) $$\begin{array}{l} R_{k}=1+\left(R_{k-1}-u_{k-1}R_{k-1}-1\right)\text{ exp}\left(-\frac{\Delta_{k-1}}{\tau_{rec}}\right) \end{array}$$

where τ_*rec*_ is the time constant for recovery from depression. The variables *u*_*k*_ and *R*_*k*_ are initialized with *u*_1_ = *U* and *R*_1_ = 1. The mean values for *U*, τ_*fac*_ and τ_*rec*_ as well as the synaptic delay depend on the type of their source and target neurons and can be found in Table 3.

**Table 3:** Population type dependent synaptic parameters; source definition as in Table 1.

	**Parameter**			**Source**	**Description**
**From/to**		**E**	**I**		
E	U	0.5 s	0.05 s	Paper	Increase of release probability with each spike
	τ_*rec*_	1.1 s	0.125 s	Paper	Time constant for depression
	τ_*fac*_	0.05 s	1.2 s	Paper	Time constant for facilitation
	*d*	1.5 ms	0.8 ms	Code	Synaptic delay
I	U	0.25 s	0.32 s	Paper	Increase of release probability with each spike
	τ_*rec*_	0.7 s	0.144 s	Paper	Time constant for depression
	τ_*fac*_	0.02 s	0.06 s	Paper	Time constant for facilitation
	*d*	0.8 ms	0.8 ms	Code	Synaptic delay

These parameters are not fixed for a given ensemble of synapses between a source population *j* and a target population *i*; instead they are drawn from a Gaussian random distribution with a standard deviation of 50% (*b*_*U*_ for *U*, *b*_*rec*_ for τ_*rec*_ and *b*_*fac*_ for τ_*fac*_), 10% (*b*_*d*_ for the delay) or 70% (*b*_*w*_ for the weight) of their mean values. As described by Häusler and Maass (2007), all negative values or values bigger than the upper bound of the range (for *U*) are replaced by values drawn from a uniform distribution between 0 and two times the mean. Note that using a truncated normal distribution leads to a different network activity with higher firing rates (data not shown).

**Figure 1:**
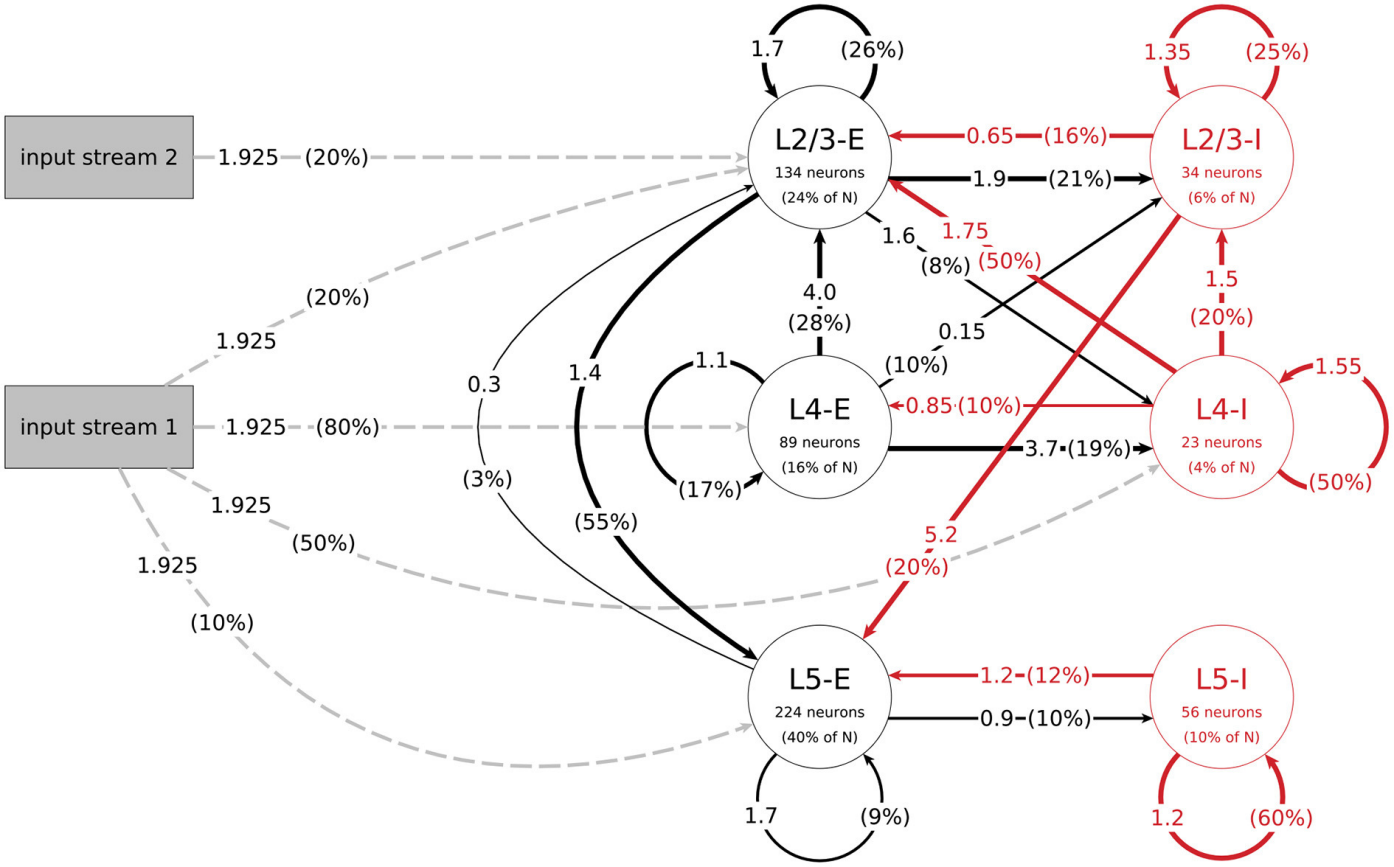
Structure of the data-based microcircuit model. The connection arrows are labeled with the connection strength (mean amplitude of PSPs in mV, c.f. parameter *A* in Table 4) and the connection probability (in parentheses). Red arrows represent inhibitory connections and excitatory connections are black. The excitatory input connections are represented as gray dashed arrows. Neuron numbers are based on a network size of *N* = 560 neurons. C.f. Figure 1, original publication (Häusler and Maass, 2007).

The mean amplitudes *A*_*ij*_ of the postsynaptic potentials for the connections between populations *j* and *i*, which are needed to calculate their mean weights, can be found in Figure 1. With this we get the value for their weight *w* by:


(27) $$\begin{array}{l} w_{ex}=\frac{A_{ij}}{\left|E_{ex}-V_{mean}\right|} \end{array}$$

for excitatory synapses and


(28) $$\begin{array}{l} w_{in}=\frac{A_{ij}}{\left|E_{in}-V_{mean}\right|} \end{array}$$

for inhibitory ones. *E*_*ex*_ and *E*_*in*_ are the excitatory and inhibitory synaptic reversal potentials and *V*_*mean*_ is the mean membrane voltage of a neuron without input. All values of the synaptic parameters can be found in Tables 3, 4.

**Table 4:** Synapse parameters.

**Parameter**	**Value**	**Source**	**Description**
*A*	see Figure 1	Paper	Mean amplitude of PSPs
*A*_*input*_	1.924779612734342 mV (1.9 mV)	Code (paper)	Mean amplitude of PSPs for input connections
*V*_*mean*_	–65 mV	Destexhe et al., 2001	Mean membrane voltage of a neuron without input
*S*_*RW*_	66,825/N (60,000/N)	Code (paper)	Scaling parameter for recurrent connections
*S*_*R*_*W*__*static*__	*S*_*RW*_/73	Experiment	*S*_*RW*_ for data-base circuit with static synapses
*S*_1_	14.85 (14)	Code (paper)	Scaling parameter for connections from stream 1
*S*_2_	36.498 (33)	Code (paper)	Scaling parameter for connections from stream 2
*w*_*ex*_	$S_{RW}\cdot \frac{A\cdot g_{L}}{\left|E_{ex}-V_{mean}\right|}$	Code	Maximum conductance for excitatory synapses
*w*_*in*_	$S_{RW}\cdot \frac{A\cdot g_{L}}{\left|E_{in}-V_{mean}\right|}$	Code	Maximum conductance for inhibitory synapses
*b*_*U*_	0.5	Paper	Factor defining the std. for the distribution of *U*
*b*_*rec*_	0.5	Paper	Factor defining the std. for the distribution of τ_*rec*_
*b*_*fac*_	0.5	Paper	Factor defining the std. for the distribution of τ_*fac*_
*b*_*d*_	0.1	Code	Factor defining the std. for the distribution of *d*
*b*_*w*_	0.7	Paper	Factor defining the std. for the distribution of *w*_*ex*_ and *w*_*in*_

The source column indicates where the value can be found, searching in the following order: main replicated paper, referenced papers, source code, own experiments. If a value was given in the paper which differs from the one used in the code, the paper value is written in parenthesis.

#### 2.1.4. Network models

**Figure 2:**
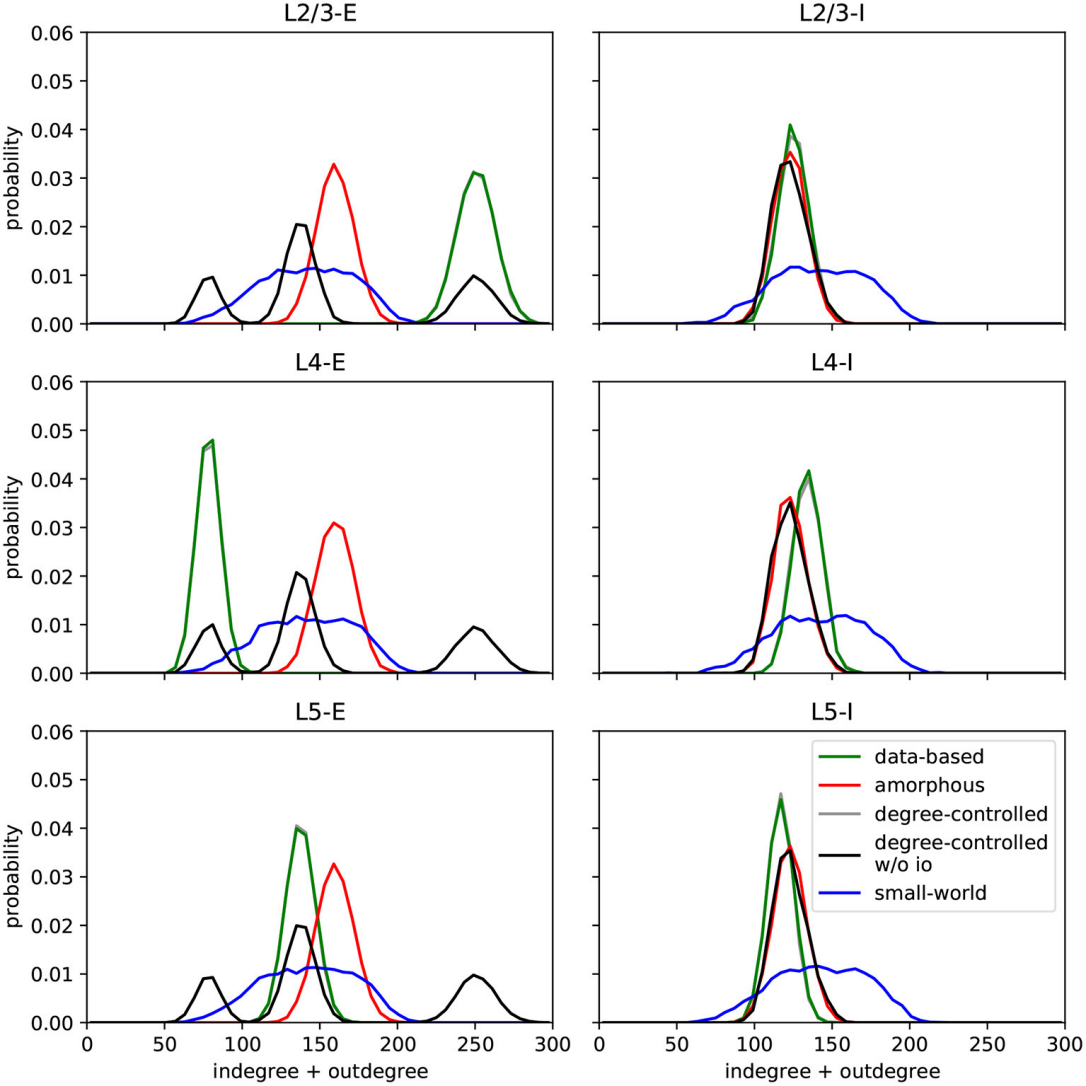
Histograms of degrees (number of incoming and outgoing connections per neuron) per population for each circuit. Values are aggregated for 100 runs with different seeds for each model. C.f. Figure 7, original publication (Häusler and Maass, 2007).

In this section, we describe the different network models implemented in the original work and in this replication. All circuits comprise 560 of the Hodgkin-Huxley neurons described above, unless otherwise stated. Another common feature shared by six of the seven circuits is that they are connected by synapses with short-term adaptation as described above. The exception is the data-based model variant with static synapses, which helps us examine the effects of synaptic dynamics on task performance. Figure 2 shows the histograms of degrees (number of incoming and outgoing synapses) for the different circuits; this serves as the first validation of our work, as they are visually indistinguishable from those presented in **Figure 7** of Häusler and Maass (2007), which is the best that can be achieved without access to the original data.

##### Data-based circuit

The data-based model consists of three layers, each divided into an excitatory and an inhibitory population. Figure 1 illustrates the network's connectivity structure; a specification of the parameters can be found in Tables 3, 4. Since the data on which the circuit is based comes from biological systems with a much larger number of incoming connections per neuron, the synaptic weights in the model are scaled up by a factor *S*_*RW*_ to obtain a reasonable network activity. In the paper, the value of this scaling factor is given as 60000/*N* (about 107 for *N* = 560), but in the published code this parameter is calculated as 66825/*N* (about 119 for *N* = 560). We use the second value in our implementation, because it gives a network activity closer to the reported one. The distribution of degrees for this connectivity model (and all following models) can be seen in Figure 2.

##### Amorphous circuit

The amorphous circuit is derived from the data-based circuit by destroying the laminar connectivity structure: for each connection, we replace the source neuron with a random neuron of the same type (excitatory or inhibitory) and also the target neuron with a random neuron of the same type (excitatory or inhibitory), whereby the new randomly selected neurons are not constrained to belong to the same layer as the ones they replace. Multiple connections between the same neuron pair are excluded. This results in a network that shares most global statistics with the data-based model: number of synapses, their pre- and postsynaptic neuron type and the distribution of all synaptic parameters such as the weights and the parameters defining the short-term dynamics remain unchanged.

##### Degree-controlled circuit

The degree-controlled circuit is also derived from the data-based circuit by scrambling its connections. However, in this network, we ensure that the number of incoming and outgoing connections (the degree) for each neuron remains unchanged. To achieve this, we randomly select two synapses whose source neurons are of the same type (excitatory or inhibitory) and whose target neurons are also of the same type, and exchange the target neurons of these synapses. We continue this procedure until none of the original connections remain. Just as in the amorphous circuit, the global statistics of the network are preserved. In addition, the number of incoming and outgoing connections per neuron is the same as in the data-based circuit.

##### Degree-controlled circuit without input or output specificity

The degree-controlled circuit without input or output specificity is derived from the degree-controlled circuit by changing the neurons to which external input is given and from which the states are read out. We implement this by randomly exchanging the layer assignations of neurons of the same type (excitatory or inhibitory) after recurrently connecting the network, but before connecting the external input streams and readouts.

##### Small-world network

As introduced by Watts and Strogatz (1998), the small-world network is one in which the underlying undirected graph has small-world properties. Such networks show a higher clustering coefficient than amorphous circuits, while keeping the average shortest path length at a comparable value. Watts and Strogatz define the local clustering coefficient of a node as the fraction of all possible connections between the node's neighbors that actually exist. It represents how close the neighborhood is to being a clique. The global clustering coefficient of a network is the average of all local clustering coefficients. The shortest path length between two nodes measures the separation of nodes and is defined as the minimum number of links required to get from one node to the other. This shortest path length is averaged over all possible node pairs in the network. We generated a small-world network using the spatial growth algorithm proposed by Kaiser and Hilgetag (2004); first we initialize the network by assigning the position (0.5, 0.5) to a random node, then we perform the following steps:

1. Take a new node and assign it a random position (*x, y*) with coordinate values drawn from the interval [0,1].
2. Connect the new node with all other nodes with probabilities defined by:
  (29) $$\begin{array}{l} P\left(u,v\right)=\beta e^{-\alpha d\left(u,v\right)} \end{array}$$
  where *d*(*u, v*) is the euclidian distance between the nodes *u* and *v*, β is a general density parameter and α is a spatial range parameter, which regulates the dependence of the connection probability on the distance.
3. Repeat steps 1 and 2 until the desired number of nodes has been reached.

By choosing α = 4 and β = 1.32 we obtain small world networks which have a clustering coefficient around 36% and a average shortest path value of about 1.75 links, comparable to those of data-based circuits.

To get the final connections for the network, we randomly assign a direction to every edge and set the weight and other synaptic parameters according to the neurons' population affiliations (see Figure 1 and Table 3). If a neuron pair belongs to two populations which aren't connected in the data-based circuit (see Figure 1), we randomly draw a weight from connection definitions for the same synapse type (excitatory or inhibitory). For example, given a connection from L5-E to L4-I, we would randomly select another excitatory connection such as L4-E to L4-I and use its weight. Since the other synaptic parameters depend only on the type (excitatory or inhibitory) of the connected neurons and not on the exact population affiliation, we can read them out from Table 3 as we did for all other connections.

##### Data-based circuit with static synapses

This network is identical to the data-based circuit, but with the dynamic synapse model replaced by static synapses. To achieve a similar network activity we have to adjust the scaling parameter *S*_*RW*_. As this value is not explicitly stated by Häusler and Maass (2007), we tuned this parameter by hand to obtain firing rates of the network as close as possible to the data-based circuit, under the condition that no population is silent. This is achieved at a value $S_{RW}^{\text{st}}=S_{RW}/73$ (see Supplementary materials for the resulting firing rate histograms of all networks).

##### Data-based circuit with random synaptic dynamics

This network is identical to the data-based circuit, except that the short-term dynamics of the data-based network's connections are scrambled. To do this, for each synapse we randomly select one of the four connection types (EE, EI, IE, II) independently of the actual source and target of the synapse. We then draw values for the parameter values *U*, τ_*rec*_, τ_*fac*_, and *d* according to the corresponding distributions for the selected connection type, with mean values as given in Table 3 and standard deviation factors as given in Table 4.

### 2.2. Tasks

Häusler and Maass (2007) implemented several different tasks to evaluate the computational performance of the different network models. Some of the tasks are based on the classification of precise spatio-temporal spike patterns and for others the circuits need to perform computations on the input firing rates of input spike trains whose spikes are generated by a Poisson process. All tasks are based on inputs given as two input streams which are connected to the network as shown in Figure 1.

Analogously to the scaling of recurrent connections by the factor *S*_*RW*_, the weight values of the synapses from these streams to the circuit are multiplied by their scaling factors *S*_1_ and *S*_2_. As can be seen in Table 4 the values given in the paper differ from the values in the code; we use the latter in our implementation.

Depending on the task, the input streams consist of either four (rate based tasks) or 40 (spike pattern classification tasks) spike trains. Per trial, 15 segments with a duration of 30 *ms* are generated, resulting in a spike train of 450 *ms* for each trial. Only the input for the retroactive spike pattern classification with fixed inter-stimulus input and the more finely resolved memory tasks, which are both further explained in the next section, are exceptions to this scheme.

**Figure 3:**
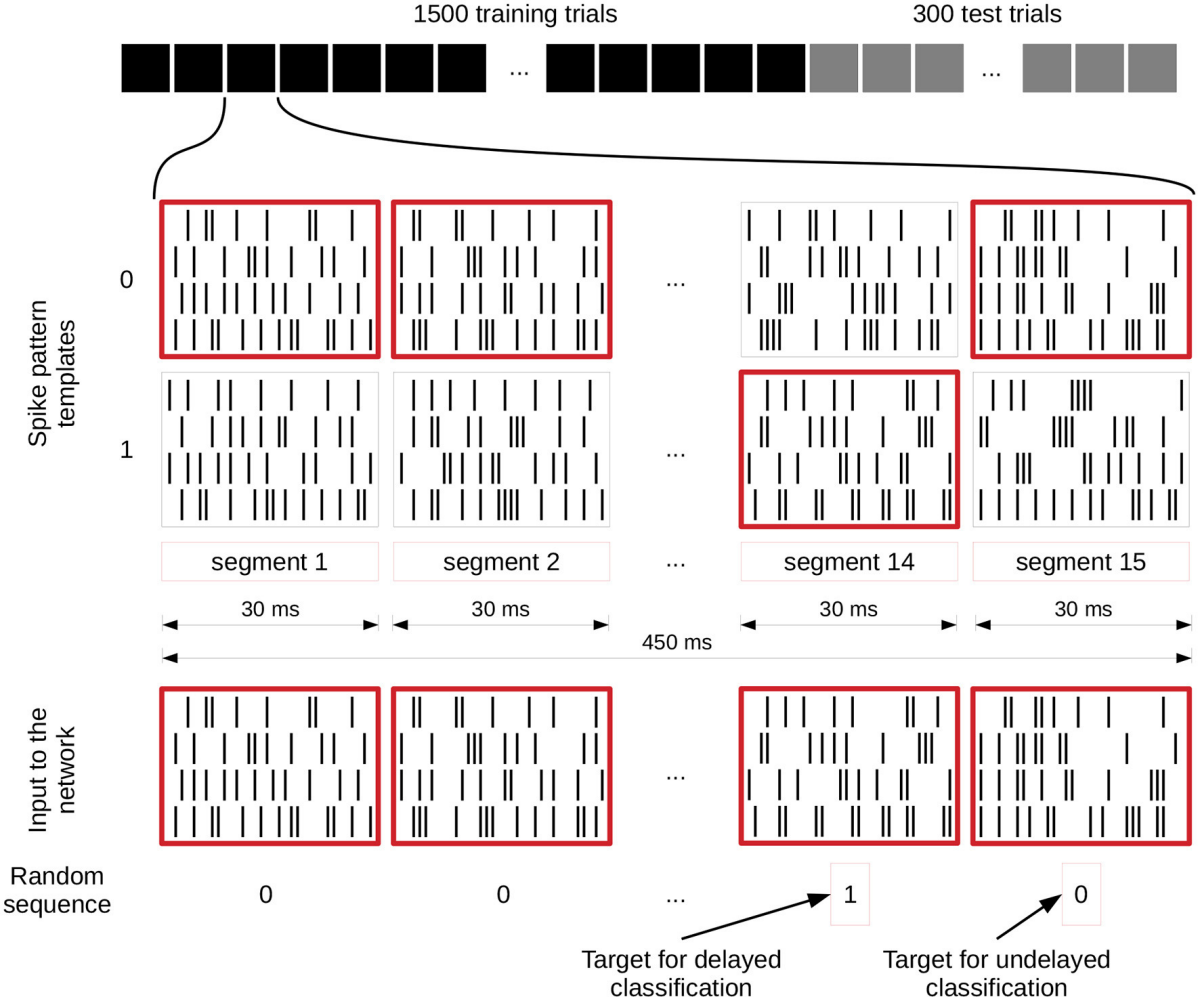
Input generation for spike pattern based tasks. The first row shows the different trials of an experiment and the spike patterns below that represent a zero or one value for each segment of a single trial. These spike pattern templates are identical for all trials. The spike patterns at the bottom are chosen based on and therefore represent the randomly generated sequence of zeros and ones underneath, which also define the target for the readout training (value of segment 14 for delayed classification and segment 15 for the undelayed classification). We give a jittered version of these spike trains to the network (jittering is not shown). C.f. Figure 4, original publication (Häusler and Maass, 2007).

Figure 3 illustrates how these input streams are generated for the spike pattern based tasks. For each segment, two different spike patterns are generated which encode either a zero or a one and the randomly generated input value (bottom row of zeros and ones in the figure) defines which of them is used in the current trial. These two possible patterns for each segment remain identical for each trial. In this way a sequence of 15 zeros and ones is translated into a set of spike trains of 450 *ms* length. In addition, each input spike is jittered by a Gaussian distribution with mean 0 *ms* and a standard deviation of 1 *ms*. We apply this jittering once per trial to the selected templates and each neuron connected to the input receives the same jittered version of the spike train. Even though some tasks are calculated based on only one of the inputs, both streams are activated and connected all the time, and the tasks are then evaluated for each input separately.

The tasks are performed by the networks using a reservoir computing approach and thus require readout neurons. We connect two different readouts to the systems: the first readout mimics an excitatory neuron of layer 2/3 and sums up the filtered spike trains of its inputs. Exactly like a normal layer 2/3 neuron, it does not receive input from all possible sources in a linked population, but is randomly connected to a subset of the units based on the corresponding connection probability. The second readout mimics an excitatory layer 4 neuron in the same way. We filter the spikes with an exponential function using a time constant of 15 *ms*. Note that inhibitory neurons are connected to the spike filtering devices with a negative weight, resulting in negative values. This is important because the readout weights are trained with a linear least squares method with non-negativity constraints. This results in non-negative readout weights and thus forces the readouts to be in accordance with Dale's principle (Eccles et al., 1954), which states that a neuron releases the same set of transmitters at all of its synapses. The states on which the readouts are trained and tested are the values of the filtered spike trains at the end of each 450 *ms* trial. A specification of the task parameters can be found in Table 5.

**Table 5:** Task and training parameters.

**Parameter**	**Value**	**Source**	**Description**
τ_*filter*_	15 *ms*	Paper	Time constant for spike filtering
*T*_*train*_	1, 500	Paper	Default number of training trials
*T*_*test*_	300	Paper	Number of test trials
*n*_*seg*_	15	Paper	Number of segments per trial
*t*_*seg*_	30	Paper	Duration of a single input segment

#### 2.2.1. Spike pattern based tasks

We implemented three main spike pattern tasks: spike pattern classification tcl_*i*_(*t*), where *i* denotes the input stream for which the classification is performed, delayed spike pattern classification tcl_*i*_(*t* − Δ*t*), and the exclusive-or task (XOR). The inputs for all of these tasks are exactly the same; the difference lies in the task-specific training of the readout weights. For the instantaneous spike pattern classification, the readout weights are trained on the prediction of the value (0 or 1) of the last segment of each trial (segment 15), whereas the target of the delayed classification is the value of the penultimate segment (segment 14), see Figure 3.

In a further set of experiments that go beyond the original study, we use a step duration of 5 *ms* instead of the standard 30 *ms* and classify the spike patterns of all 15 segments. As these tasks are all based on only one input, we evaluate them for both input streams separately.

In contrast to this, the XOR task is computed based on the value of the last segment of both input streams. To evaluate the task performances we use a threshold of 0.5 to fix the readout predictions to values of zero or one, and calculate the kappa coefficient between the target output and the predicted output. The kappa coefficient is calculated as:


(30) $$\begin{array}{l} \kappa =\frac{P_{0}-P_{C}}{1-P_{C}} \end{array}$$

where *P*_0_ is the agreement between the target and the observed prediction and *P*_*C*_ is the chance agreement.

In addition to these three task types, we also implemented the retroactive spike pattern classification with fixed inter-stimulus input, which Häusler and Maass (2007) use to evaluate the training convergence in dependence on the number of training trials. The task is to classify spike patterns consisting of four spike trains with a duration of 100 *ms* after an intervening fixed spike pattern of 100 *ms* was given to the network in every trial. We implemented this by setting the segment duration to 100 *ms*, the number of segments per trial to two, the input dimension to four, the second input value of every trial to zero and the first input value as the target for the readout. We also disabled spike jittering for the fixed spike trains between the target stimuli.

#### 2.2.2. Firing rate tasks

In addition to the spike pattern based tasks, computational tasks were also defined using time varying firing rates. The structure of the input streams is similar to the one used in the previously described tasks and the visualization in Figure 3. However, instead of taking one of two pre-generated spike patterns, we define a target firing rate between 15 and 25*spks*/*s* for each of the 15 segments in both input streams and generate the spike trains based on this rate. The firing rate of the last 15 *ms* of each trial is used as the target for the readouts. To avoid errors resulting from a division by zero, we ensure that at least one spike is placed in this last 15 *ms* window of the input streams. The tasks we implemented are the quotient of the two input streams *r*_1_/*r*_2_ and the square of their difference ${\left(r_{1}-r_{2}\right)}^{2}$. As the kappa coefficient can't be used for these analog prediction tasks, we evaluate the performance on the basis of the Pearson correlation coefficient between the prediction and the target.

### 2.3. Simulation and analysis framework

We simulate the spiking neural network experiments with a timestep of 0.2 *ms* using NEST 3.0 (Hahne et al., 2021). Since the neuron model we describe above is not included in NEST, we implement it using NESTML 4.0.0 (Babu et al., 2021). For all other data analysis and plotting we use Python 3.8.8 and a modified version of the Functional Neural Architectures library (Duarte et al., 2021).

## 3. Results

### 3.1. Network activity

**Figure 4:**
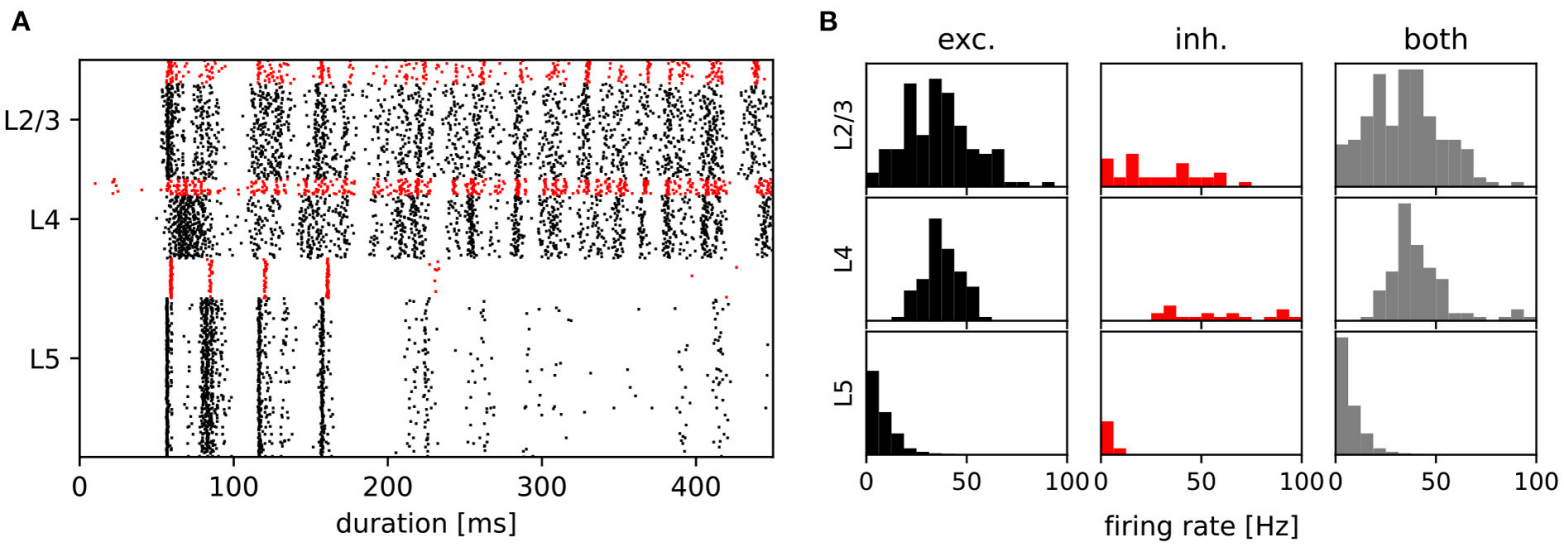
Activity of the data-based circuit. **(A)** Raster plot for the data-based circuit after input stream two was activated at 100 *ms*. Excitatory neurons are black and inhibitory neurons are red. **(B)** The corresponding firing rate histograms for each population and layer. C.f. Figures 2B,C, original publication (Häusler and Maass, 2007).

After establishing that the degree distributions of the various networks were visually indistinguishable from the published distributions (see Figure 2), we then examined the activity of the data-based network. Figure 4A shows a raster plot for the network with input stream two becoming active at 100 *ms*, and Figure 4B provides the corresponding firing rate histograms for the six populations and, combined, the three layers (c.f. Figure 1). These plots can be compared with Figures 2B,C of the original publication.

Note that whereas the firing rate histograms in Figure 4B are very similar to those shown in the original paper, the raster plot in Figure 4A exhibits some discrepancies. Most notably, the latency of network activity is longer in our implementation than in the original. Only a few inhibitory layer 4 neurons show earlier activity, and although both figures are based on trials of only 450 *ms*, this behavior is consistent in our experiments. Less consistent is the measured firing rate of layer 5. In contrast to the original study, which reports a stable firing rate of around 8.5 *spks*/*s* in this layer, we observe a range of firing rates between 3 and 9 *spks*/*s* for differently seeded runs. A possible explanation for these discrepancies is that in the original code, the values of *E*_*M*_ and *V*_*m*_ are transformed into a different simulation voltage range to compute the non-inactivating K^+^ current *I*_*M*_. For this transformation, values of −70 *mV* for the resting potential and −40 *mV* for the threshold potential were used, rather than the values used in the rest of the study (−80 *mV*, −30 *mV*, see Table 1). In our implementation, we elected not to include these transformations in the neuron model, as we could determine neither a biological basis nor a computational advantage for so doing; as shown in the following sections, a qualitative reproduction of the task performances is achieved without such transformations.

### 3.2. Task performance for the circuit variants

**Figure 5:**
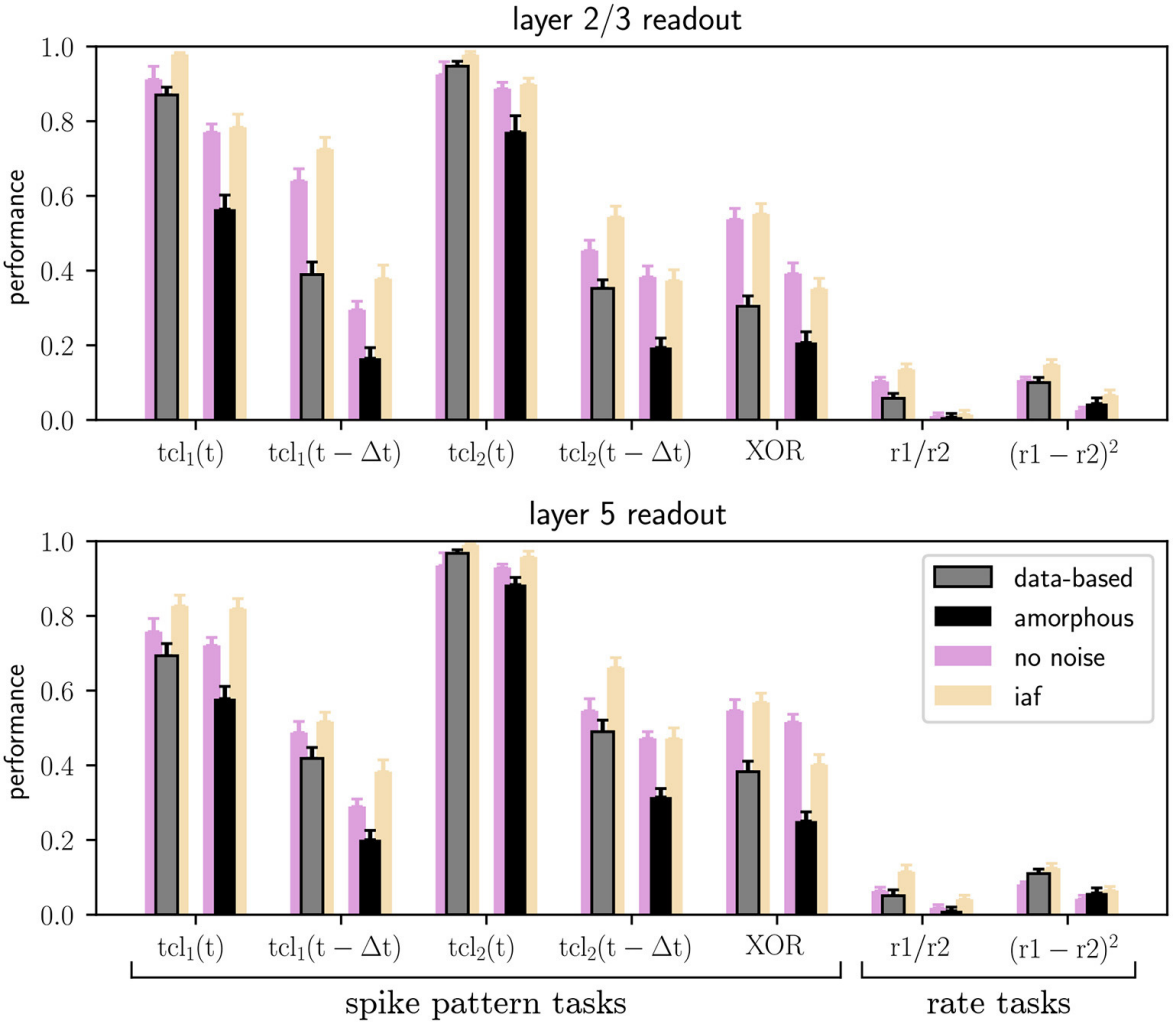
Performance of trained linear readout neurons in layers 2/3 and layer 5 for the classification tasks on spike patterns and computations performed on the rates of the two input streams (see Section 2.2), both for data-based laminar microcircuit models (gray bars) and for the amorphous control circuits (scrambled laminar structure; black bars). Light purple bars represent the results for networks with neurons without conductance noise and light orange bars networks consisting of integrate-and-fire neurons. Error bars are the standard errors of mean. All values are averaged over 20 runs. C.f. Figure 5, original publication (Häusler and Maass, 2007), likewise averaged over 20 runs.

Figure 5 shows the results of the seven main tasks for the data-based and the amorphous circuits. Although the performance values are not identical to the ones in the original study, the values are close and qualitatively reproduce the key finding that the data-based circuit outperforms the amorphous control circuit in every task. The main difference between our results and those reported in the original study is our comparatively low performance at rate based tasks and the delayed spike pattern classification of input stream one (for both circuits).

**Figure 6:**
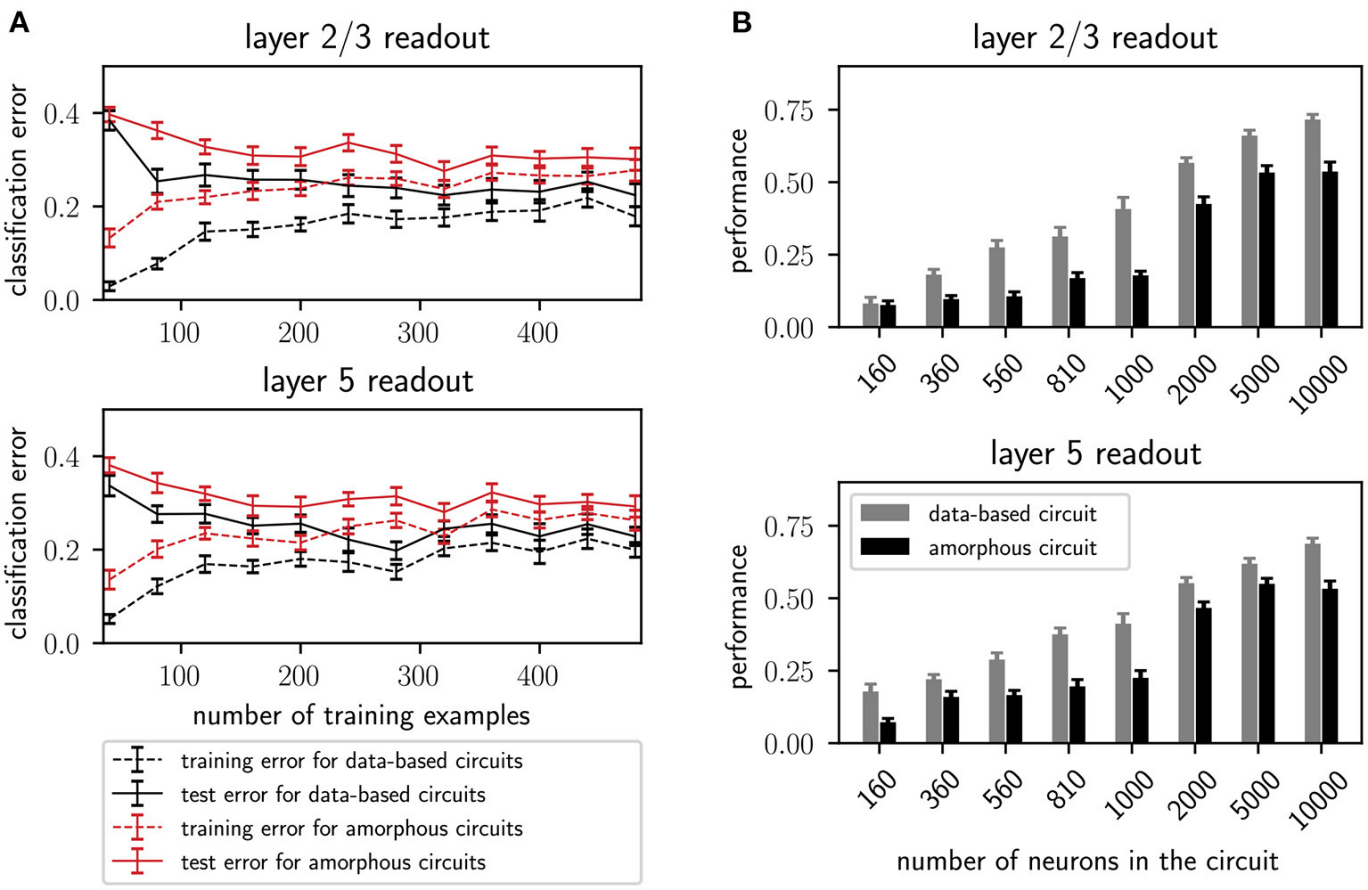
**(A)** Training and testing error of readouts from data-based and amorphous circuit models as functions of the size of the training set. 300 trials are always used for testing. Error bars indicate the standard error of means. Values are averaged over 30 runs. C.f. Figure 8, original publication (Häusler and Maass, 2007), averaged over 20 runs. **(B)** Performance (kappa coefficient) on the XOR task of projection neurons in layers 2/3 and layer 5 for different circuit sizes, with and without a data-based laminar structure. All values are averaged over 30 runs. Error bars indicate the standard error of the means. C.f. Figure 6, original publication (Häusler and Maass, 2007), averaged over 10 runs.

Additionally, Figure 6A shows the performance of the data-based and amorphous circuit for the retroactive spike pattern classification task with fixed inter-stimulus input for different numbers of training examples. The original study does not specify which input stream was used to generate the corresponding figure in their work (**Figure 8**); we therefore tested both of them. Our experiments show more similar results for input stream one, and so we use those results as the basis for Figure 6A. As in the original study, the data-based circuit has a lower test and training error than the amorphous circuit for all sizes of training set. Taken together, Figures 5, 6A support the argument put forward by the original study that a laminar structure has a positive effect on the computational performance of a circuit.

The quantitative performance measures for all circuits (Section 2.1.4) and all tasks (Section 2.2) can be found in Table 6. These results can also be expressed as percentage difference from the performance of the data-based circuit; this analysis is given in Table 7.

**Table 6:** Performance measures for all networks and all tasks.

**Tasks/circuits**	**Data-based**	**Amorphous**	**Small-world**	**DC**	**DC (no io)**	**Random dynamics**	**Static synapses**
tcl_1_(t) (L23)	**0.87**	–0.305	–0.17	–0.106	–0.235	–0.117	0.013
tcl_2_(t) (L23)	**0.947**	–0.175	–0.171	–0.097	–0.168	–0.44	–0.067
tcl_1_(t) (L5)	**0.693**	–0.115	–0.145	–0.106	–0.049	–0.407	–0.069
tcl_2_(t) (L5)	**0.968**	–0.084	–0.117	–0.065	–0.191	–0.469	–0.159
tcl_1_(t − Δ*t*) (L23)	0.389	–0.224	–0.024	–0.061	–0.131	**+0.021**	–0.056
tcl_2_(t − Δ*t*) (L23)	0.352	–0.158	–0.02	**+0.071**	–0.081	–0.185	–0.091
tcl_1_(t − Δ*t*) (L5)	**0.418**	–0.217	–0.073	–0.117	–0.101	–0.136	–0.258
tcl_2_(t − Δ*t*) (L5)	**0.49**	–0.175	–0.119	–0.007	–0.187	–0.259	–0.196
XOR (L23)	**0.304**	–0.097	–0.145	–0.053	–0.114	–0.174	–0.061
r1/r2 (L23)	0.058	–0.05	**+0.083**	+0.043	+0.076	+0.073	–0.007
(r1 − r2)^2^ (L23)	0.1	–0.056	+0.026	**+0.028**	+0.012	+0.006	–0.013
XOR (L5)	**0.383**	–0.132	–0.206	–0.102	–0.198	–0.206	–0.095
r1/r2 (L5)	0.051	–0.04	+0.069	+0.064	**+0.116**	+0.029	–0.025
(r1 − r2)^2^ (L5)	0.11	–0.052	**+0.026**	+0.017	+0.014	–0.02	–0.067
Memory	**0.43**	–0.183	–0.07	–0.001	–0.147	–0.17	–0.135
Nonlinear	**0.168**	–0.072	–0.025	–0.001	–0.016	–0.049	–0.045
Other	**0.938**	–0.162	–0.144	–0.083	–0.196	–0.373	–0.093
All	**0.463**	-0.129	-0.072	-0.024	-0.105	-0.176	-0.084

Spike based tasks are evaluated with the kappa coefficient and rate based tasks with the correlation coefficient. The data-based column gives the absolute value and the other columns show the difference from this value. The best performance per task/row is marked in bold. Gray/blue shading denotes tasks from the categories memory/nonlinear. All values are averaged over 20 runs (10 runs in original paper).

**Table 7:** Performance measures for all control networks and all tasks expressed as average difference (in percent) from the performance of the data-based circuit.

**Taks/circuits**	**Amorphous**	**Small-world**	**DC**	**DC (no io)**	**Random dynamics**	**Static synapses**
tcl_1_(t) (L23)	–35.1	–19.6	–12.2	–27.0	–13.4	1.5
tcl_2_(t) (L23)	–18.5	–18.1	–10.2	–17.8	–46.5	–7.1
tcl_1_(t) (L5)	–16.6	–20.9	–15.3	–7.2	–58.7	–10.0
tcl_2_(t) (L5)	–8.6	–12.1	–6.6	–19.6	–48.4	–16.4
tcl_1_(t − Δ*t*) (L23)	–57.5	–6.2	–15.6	–33.8	**5.4**	–14.4
tcl_2_(t − Δ*t*) (L23)	–44.9	–5.9	**20.1**	–23.1	–52.6	–25.9
tcl_1_(t − Δ*t*) (L5)	–52.0	–17.5	–28.1	–24.3	–32.5	–61.8
tcl_2_(t − Δ*t*) (L5)	–35.7	–24.3	–1.4	–38.2	–52.9	–40.0
XOR (L23)	–31.9	–47.8	–17.6	–37.5	–57.4	–20.2
r1/r2 (L23)	–86.8	**142.6**	**74.1**	**130.8**	**126.5**	–11.2
(r1 − r2)^2^ (L23)	–56.1	**26.3**	**28.2**	**11.8**	**6.3**	–12.7
XOR (L5)	–34.5	–53.9	–26.7	–51.8	–53.9	–24.8
r1/r2 (L5)	–79.2	**136.1**	**126.4**	**227.8**	**57.4**	–48.6
(r1 − r2)^2^ (L5)	–47.1	**23.7**	**15.5**	**12.3**	–18.0	–61.2
Memory	–42.5	–16.4	–0.2	–34.1	–39.7	–31.3
Nonlinear	–42.5	–14.7	–0.3	–9.5	–29.0	–26.6
Other	–17.3	–15.3	–8.8	–20.9	–39.8	–9.9
All	–27.9	–15.5	–5.2	–22.6	–38.1	–18.2

Positive values indicating a performance improvement with respect to the data-based circuit are marked in bold. Gray/blue shading denotes tasks from the categories memory/nonlinear. All values are averaged over 20 runs. C.f. Table 2 in original publication (Häusler and Maass, 2007).

The last four rows of both tables show results averaged over both readouts and over a category of tasks. The *memory* row averages over all tasks for which the networks need to memorize earlier inputs [tcl_1_(*t* − Δ*t*) and tcl_2_(*t* − Δ*t*)], the *nonlinear* row averages the results over the tasks based on nonlinear computations (XOR, *r*1/*r*2 and (*r*1 − *r*2)^2^) and the *other* row summarizes all other tasks [tcl_1_(*t*) and tcl_2_(*t*)]. The last row of the tables averages the results over all tasks. In the original paper only the last four rows of Table 7 were presented (Table 2 in Häusler and Maass, 2007).

According to the averaged results, the data-based circuit outperforms all other circuits on all tasks. Here we have broad agreement with the original study, in which only the degree controlled network had slightly superior performance in two task categories.

Examining the disaggregated data in Table 7, we observe that there are 17 instances where a control circuit exhibited superior performance to the data-based circuit. In particular, most networks surpass the data-based circuit on all rate-based tasks. However, as Table 6 shows, the performance for these tasks is very low for all networks, which means that even a small absolute increase in the correlation coefficient results in a substantial percentage increase. We therefore consider this partial contradiction of the original study to be neuroscientifically uninteresting.

In addition to the rigorous analysis of the effect of circuit connectivity, the original study also considered the influence of network size by increasing the number of neurons within each population of the circuit. Figure 6B shows the dependence of the XOR task performance as a function of the number of neurons in the circuit. As with the original study, our results show a systematically better performance for the data-based circuit over the amorphous circuit, and an increase in performance for both circuits types with increasing circuit size up to 5, 000 neurons. While the data-based network still benefits from increasing the network size to 10, 000, the performance of the amorphous circuit reaches its maximum value at 5, 000 neurons. This effect could not be observed in the original study, as the maximum size of network examined was 1, 000 neurons.

### 3.3. Robustness to neuron model simplifications

**Figure 7:**
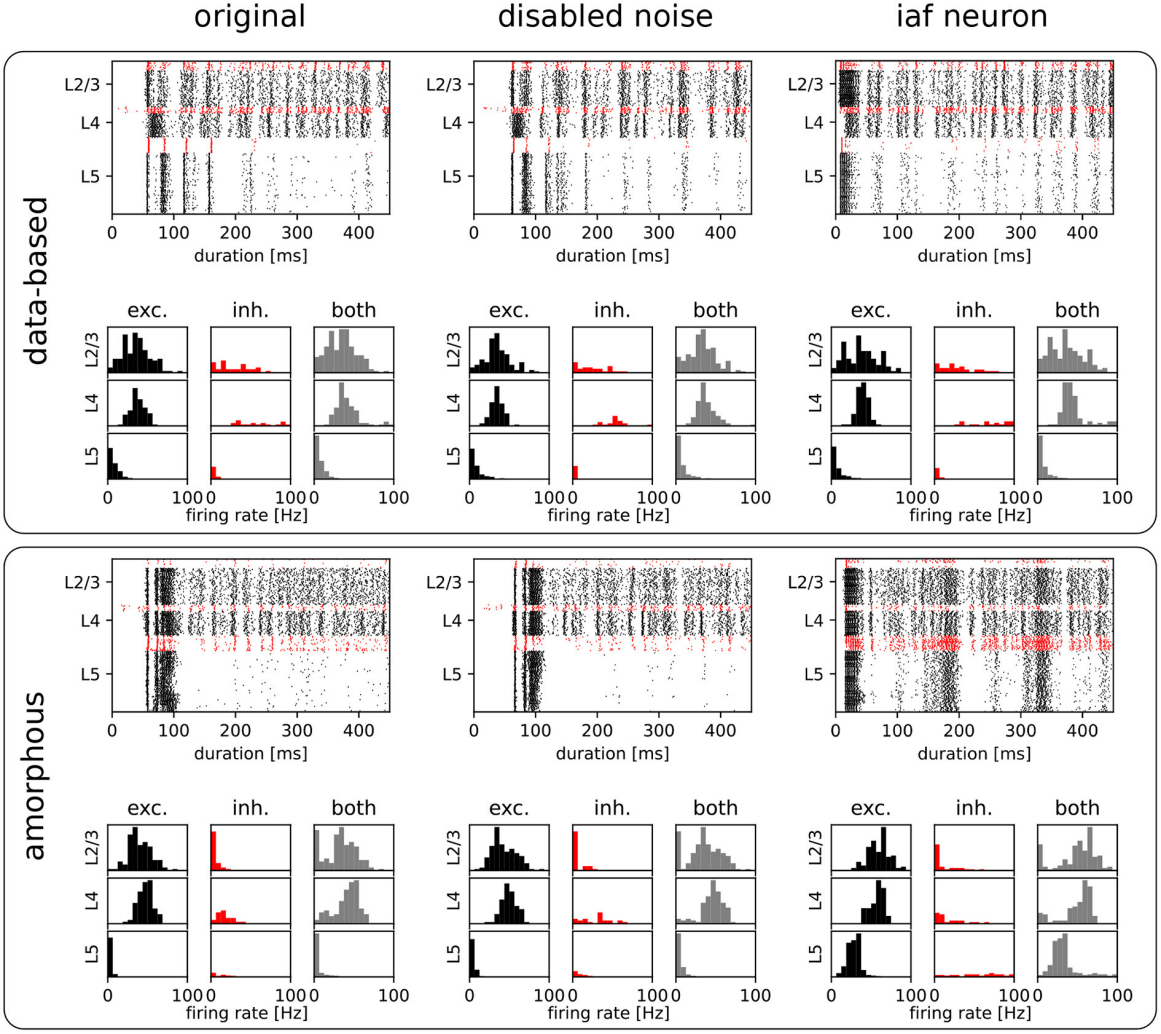
Raster plots and firing rate histograms for the data-based and amorphous circuits for the three different neuron types (original: Hodgkin-Huxley neurons that were used in the original publication, disabled-noise: Hodgkin-Huxley neurons without intrinsic conductance noise, iaf neuron: integrate-and-fire neurons). The spikes of the inhibitory populations are colored red, while those of the excitatory populations are shown in black. As in Figures 2B,C, original publication (Häusler and Maass, 2007), for the raster plots input stream two starts at 100 *ms*.

The original study demonstrated the computational benefits of lamina-specific connectivity using a fairly complex neuron model. We therefore hypothesize that the details of the neuron model are not relevant to this key finding. To test this hypothesis, we examine the robustness of our dynamical and computational results to variations in the neuron model (see Section 2.1.2). First, we consider the intrinsic noise mechanism. As shown by the raster plots and firing rate histograms in Figure 7, the firing activity in the networks does not change significantly for the data-based and amorphous circuits in the absence of intrinsic noise. Moreover, we observe that the data-based connectivity structure is still superior to all other connectivity patterns in all task types, which Figure 5 (light purple bars) illustrates for the comparison with the amorphous circuit (see Supplementary materials for the summarized performance measures for all circuit types). Figure 5 also shows that networks without noise (both data-based and amorphous circuits) perform slightly better than their noisy counterparts in most of the tasks performed, and even considerably better in the nonlinear XOR task.

Second, we reduce the neuron model from a Hodgkin-Huxley to a much simpler integrate-and-fire neuron model. To obtain firing rate ranges in the data-based circuit as close as possible to those of the network with Hodgkin-Huxley neurons, we adjust *V*thresh of the integrate-and-fire neuron model to a different value for each population (see Section 2.1.2 for the parameter values and Supplementary materials for the firing rate distributions). The top right part of Figure 7 shows the corresponding raster and firing rate plots.

Also among the circuits consisting of integrate-and-fire units, the data-based network has the best task performance (see Supplementary materials for the summarized performance measures). Moreover, Figure 5 shows similar results for the Hodgkin-Huxley neural networks without noise (light purple bars) and the iaf circuits (light orange bars), with the XOR task values of the amorphous circuits showing the most noticeable difference.

We conclude that these results confirm our hypothesis that the superiority of the data-based connectivity structure does not depend on the specifics of the neuron model. Moreover, they reveal that a reduction in complexity even leads to an increase in performance on the tasks conducted. We hypothesize that the dynamics of the Hodgkin-Huxley model, which are much more intricate than those of integrate-and-fire neurons, may effectively act as an additional noise source that reduces task performance.

### 3.4. Detailed memory tasks

**Figure 8:**
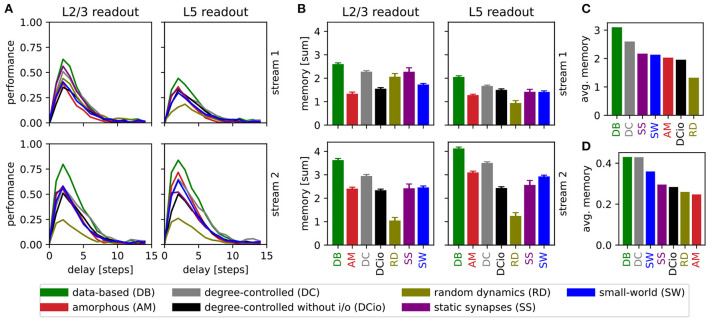
Results of retroactive spike pattern classification tasks for all network types. **(A)** Performances (kappa coefficient) for the classification of spike patterns with a duration of 5 *ms* at different delays, separated by input stream and readout (averaged over 40 trials). **(B)** Bars representing the sum of task performances over all delays for the same task as in **(A)**. Error bars represent the standard error of mean. **(C)** Values from **(B)** averaged across all input streams and readouts. **(D)** Averaged results of the delayed classification of 30 *ms* spike patterns (data of *memory* row in Table 6).

To get further insight into the memory capabilities of the networks, we devised a modification of the retroactive spike pattern classification task, namely reducing the step size from 30 *ms* to 5 *ms* and classifying the spike patterns of all 15 segments (see Section 2.2). This gives our view on retroactive spike pattern classification a six times higher resolution with one data point for every 5 *ms* interval instead of only every 30 *ms*, allowing us to determine the memory profile for each circuit variant. Our results for the more detailed memory tasks are summarized in Figure 8.

We observe that all network variants require some processing time to reach their peak performance, see Figure 8A. For all combinations of network, input stream, and readout location, the maximum kappa coefficient is reached after a delay of two steps (10 *ms*), i.e., the networks have the greatest accuracy in identifying the stimulus inserted two steps before the current one. The only exception is the layer 5 classification of input stream 1 of the data-based network with random synaptic dynamics, which reaches its maximum after a delay of three steps (15 *ms*). However, in general the performance increases steeply up to delay 2 (10 *ms*) and then decreases more slowly until all circuits reach a value close to zero at delay 10 (50 *ms*).

Notably, the performance of the undelayed classification is worse than that of the short-term delayed classification, in contrast to the results presented in Figure 5 for a step of 30 *ms*. This can be understood by considering that the networks need more than 5 *ms* to process the input and generate an informative response from the few neurons on which the readouts are based. One reason for this is synaptic delays, since for example excitatory-to-excitatory synapses already require an average of 1.5 *ms* to transmit a single spike from a presynaptic to a postsynaptic neuron. With the longer step of 30 *ms*, the network has plenty of time to respond informatively to the undelayed stimulus, whereas the effects of the previous stimulus have already faded considerably. Likewise, the longer step duration provides greater possibilities for readout weights to be learned that accurately distinguish between stimuli, resulting in a better peak performance for the 30 *ms* task.

The heights of the bars in Figure 8B indicate the sum of the values for all delays in Figure 8A. This illustrates that not only does the peak performance of the circuit with data-based connectivity surpass that of all other systems at the optimal delay, as shown in the previous line graph, but also that a more general view encompassing the task results for all delays reveals the superiority of this circuit. As the data-based circuit does not retain stimulus information for longer than the other circuits, we conclude that its superior performance must be due to the laminar connectivity enabling it to generate more distinct representations of the input stimuli.

Figure 8C generalizes this view even further by averaging the results shown in Figure 8B over the input stream and readout location, and sorting the networks by performance. Here, the degree-controlled circuit follows the best-performing data-based circuit and the network with random synaptic dynamics has the lowest result. The next four systems (SS, SW, AM, and DCio; Figure 8C) are comparatively at the same level with only minor differences. Although one can not directly compare the averaged sum over multiple delays with the performance based on only a single delay, it is interesting to note that this graph does not display the same order as the results of the previous memory tasks with the longer step of 30 *ms* shown in Figure 8D and Table 6. While in both cases the data-based circuit is best and the degree-controlled circuit second best, the difference is much smaller for the original memory tasks and the order of the remaining networks is different. For both step durations, the amorphous and the degree-controlled circuit without input and output specificity show results at similar levels to the network with static synapses. For the 30 *ms* memory task type the small-world network performs comparatively better than this group, positioning itself in third place, whereas for the detailed memory tasks with 5 *ms* resolution, the network with random synaptic dynamics performs noticeably worse than the other circuits.

Clearly, the stimulus step duration has an effect on the computational capabilities of the networks and can move a network's peak performance to different delays. Moreover, the ordering differences in Figures 8C,D suggest that the optimal step duration for a network depends on the connectivity structure. However, the good performance of the degree-controlled circuit and especially the data-based circuit for both step durations tested show that both of these systems have a lower dependence on this duration parameter and can more robustly handle stimuli of different lengths.

## 4. Discussion

### 4.1. Replicability

Our results demonstrate that we could replicate the circuits of the original study and both confirm and strengthen their key findings. However, we encountered significant challenges during this process and it would have been essentially impossible if we had only had the paper as a source of information. As can be seen in the parameter Tables 1, 3, 4, several of the neuron and synapse parameters were not given in the paper, or a different value was reported than was used in the code. For example, only by examining the code could we determine the specification of the synaptic delay, namely that it is drawn from a random distribution with a mean value that depends on the connection type (EE, EI, IE, and II).

Likewise, the synaptic time constants were only given in the code and the parameterization of the neuron ion channels showed some discrepancies. In addition to minor differences in the parameters of the Na^+^ ion channel, there was greater variation in the definition of the K^+^ ion channel, which is responsible for the current *I*_M_. Häusler and Maass (2007) provide peak conductance densities for the different ion channels and a single membrane area, which can be used to calculate the peak conductance of every channel. Examining the code reveals that for the standard sodium and potassium ion channels, the reported membrane area is used to calculate the conductivity, however it is based on a different (and unreported) area value for the other potassium ion channel (responsible for *I*_M_). In addition, the conductance density of the latter channel is twice as large as that stated in the paper and different factor *r* was used in equations 15 and 16 defining the channel dynamics, which is a deviation that is easy to overlook. These differences in parameters significantly alter the activity of the network and led us to disable the M ion channel in early experiments to obtain approximately comparable network responses.

Besides the difficulty in specifying basic synaptic and neuronal parameters, the scaling parameters of the synaptic weights *S*_RW_, *S*_1_ and *S*_2_ also caused some problems. All of them were different from the values reported in the paper and it was hard to track them down in the MATLAB code, because the final scaling parameter values were not set directly, but defined by somewhat convoluted calculations distributed over multiple source code files. This was a particularly challenging example of a general problem: as the code was not executable due to its age, it was not possible to simply output the final values of variables, or examine the parameters and dynamic variables of the neurons and synapses. Instead, calculations had to be painstakingly reconstructed by analyzing the source code and replicating the logic.

As a final example of the nature of the replication challenges, the input scaling parameters in the code are approximately—but not exactly—1,000 times smaller than given in the paper, because the input weight to be scaled is approximately—but not exactly—1,000 times bigger than reported. In contrast to the weights inside the network, which are defined in the code as amplitudes of post-synaptic potentials in the same way they are given in the paper, the input weights are defined as a post-synaptic current of 30 *nA*. This results in the following PSP-amplitude:


(31) $$\begin{array}{l} PSP_{\text{input}}=\frac{PSC_{\text{input}}}{g_{\text{L}}}=1.924779612734342V \end{array}$$

instead of the reported 1.9 mV. However, the combination of these differences results in a scaled input weight with the same order of magnitude as the one reported in the paper.

These hurdles to replicating the paper provide a good demonstration of the argument presented in Pauli et al. (2018): whereas provision of source code is the absolute minimum requirement for replicating a study in computational neuroscience, the process is rendered much simpler if appropriate care is taken with the code implementation, e.g., writing modular, encapsulated, well-commented code with separation of parameters and program logic. Moreover, many of the hurdles we encountered would have been substantially reduced if the code had been executable. To foster reproducibility, we therefore recommend (again following Pauli et al., 2018) that models should not be expressed in homebrewed code, as this is unlikely to be maintained. Instead, developing the model using a simulator that is actively developed by a community reduces the maintenance load and increases the likelihood that the model will remain accessible, executable, and part of the scientific discourse for years to come.

## 5. Conclusion

We analyzed how the lamina structure of a cortical column model affects the computational capabilities of spiking neural networks. In a first step, we replicated the models and experiments described by Häusler and Maass (2007). Although we did not get identical network activity and task results, the activity results and degree histograms are close enough to demonstrate that the replication was successful. Our findings on the tasks defined in the original study confirm their key result, that the degree distribution exhibited by laminar structure of the data-based circuit confers a computational advantage over circuits with modified connectivity patterns that destroy the laminar connectivity whilst maintaining the global statistics of the network. We reach this conclusion by training readout weights to solve tasks based on spike patterns and firing rates that require linear and non-linear computations on two separate input signals and the memorization of prior information.

The microcircuit model at the heart of the original study shares many properties with biological microcircuits. In addition to its data-based structure, it consists of Hodgkin-Huxley neurons with different ion channel dynamics and a conductance-based background noise mechanism, and its synapses exhibit short-term plasticity. For further biological plausibility, its readouts receive only inputs restricted to layer 2/3 and layer 5 specific connections. The readout weights observe Dale's principle (Eccles et al., 1954): excitatory neurons contribute only positive values to the activity function of the readout neuron, and inhibitory neurons only negative values.

Given the superior performance of the data-based circuit, we formulated the hypothesis that the results should be robust with respect to the specifics of the neuron model. We extended the analysis of the original study by decreasing the complexity of the neuron model, first removing the intrinsic noise and then reducing the dynamics to that of an integrate-and-fire neuron. The results confirmed our hypothesis that neuron model details were not important to the key result: in both cases the data-based circuit continued to exhibit superior performance over all other variants. Our results also rule out the possibility that a complex neuron model is necessary for the data-based circuit to reach a good performance, since the two simpler neuron types tested outperform it in the majority of tasks. Likewise, we found that relaxing the biologically motivated restriction on the readout weights increases performance (data not shown).

To obtain a higher temporal resolution in the examination of the memory capabilities of the circuit variants, we additionally extended the original analysis to include retrospective classification of spike patterns of much shorter segments with a duration of 5 *ms* instead of 30 *ms*, and classifying all of the segments rather than just the last two. Here we observe a stereotypical memory profile for all circuit types, where the data-based circuit beats the other networks in peak performance and summed reconstruction capability across all delays, with comparable performance for the degree-controlled circuit. However, there is no significant difference between the various networks when comparing the maximum delay up to which the signal can still be at least partially reconstructed. Thus, we conclude that the advantage of the laminar connectivity structure lies primarily in the clarity of the internal representation rather than in significantly longer information retention. These results also highlight the characteristic time scale of the input as a relevant parameter for determining the computational capacities of a spiking neural network.

In future work, we will use our NEST implementation of the data-based microcircuit model, which is now freely available to all researchers, to lay the groundwork for further experiments to investigate the computational properties of cortical columns and to make quantitative comparisons with alternative microcircuit models. In this context it would also be reasonable to tune the network to obtain biologically more realistic long-tailed firing rate distributions with a mean below 1 spks/s instead of the comparatively high activity currently exhibited by the model (about 40 spks/s for layers 2/3 and 4). For the simplified network with integrate-and-fire neurons, this can probably be achieved by adjusting the firing threshold per population based on *in-vivo* data rather than using the activity of the original model as a basis. Similarly, for the network with Hodgkin-Huxley neurons, it is likely that population-level tuning of neuron parameters and probably adjusted scaling of recurrent weights will be required to achieve the intended firing rates.

From here it is also possible to add other biological details such as additional or different plasticity mechanisms, or to investigate the computational capacities of larger networks using this microcircuit as a basic building block for systems representing the meso- or macroscopic level. This could be achieved, for example, by adjusting the weight scaling parameters along with the network size and connecting multiple differently parameterized instances of the microcircuit using inter-area connectivity that is based on experimental findings.

## Data availability statement

The original contributions presented in the study are included in the article/Supplementary material, further inquiries can be directed to the corresponding author.

## Author contributions

TS, RD, and AM designed the study and performed the analysis. TS re-implemented the model, performed the simulations, and created the figures. All authors co-wrote the manuscript. All authors contributed to the article and approved the submitted version.

## Funding

This project was funded by the Helmholtz Association Initiative and Networking Fund under project number SO-092 (Advanced Computing Architectures, ACA), the Excellence Initiative of the German Federal and State Governments (ERS SDS005), and the Joint Lab Supercomputing and Modeling for the Human Brain. Open access publication funded by the Deutsche Forschungsgemeinschaft (DFG, German Research Foundation)-491111487.

## Conflict of interest

The authors declare that the research was conducted in the absence of any commercial or financial relationships that could be construed as a potential conflict of interest.

## Publisher's note

All claims expressed in this article are solely those of the authors and do not necessarily represent those of their affiliated organizations, or those of the publisher, the editors and the reviewers. Any product that may be evaluated in this article, or claim that may be made by its manufacturer, is not guaranteed or endorsed by the publisher.
