# Supplementary material for: Characteristic columnar connectivity caters to cortical computation: Replication, simulation, and evaluation of a microcircuit model
Source: Front Integr Neurosci. 2022 Oct 3;16:923468. doi: 10.3389/fnint.2022.923468 (PMC9615567; doi:10.3389/fnint.2022.923468)
Supplement: Supplementary file 1 [file Data_Sheet_1.pdf]

Supplementary Material

1 SUPPLEMENTARY TABLES

1.1 Figures

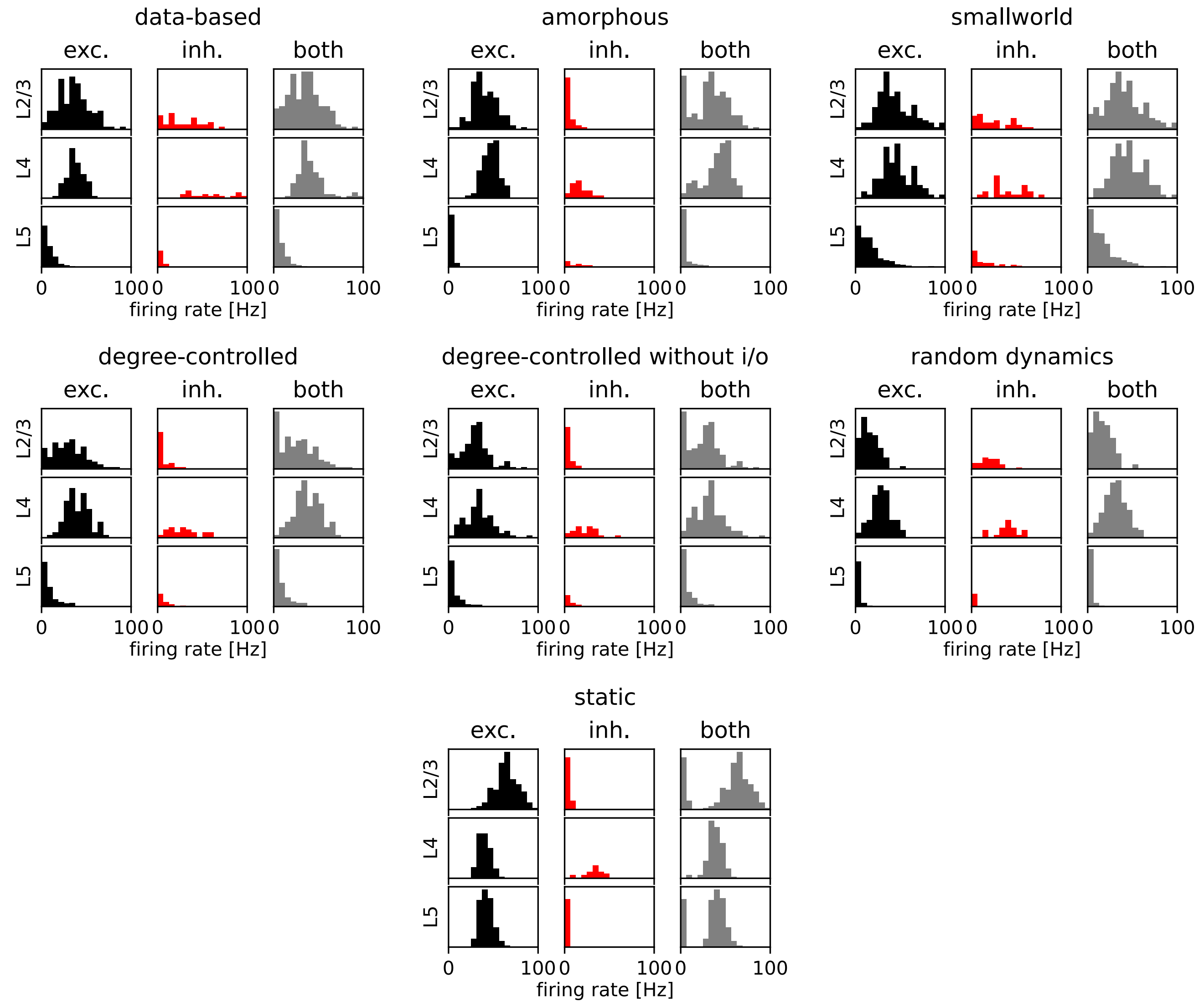

Figure S1. Firing rate histograms of the control circuits consisting of Hodgkin-Huxley neurons with conductance noise.

1.2 Tables

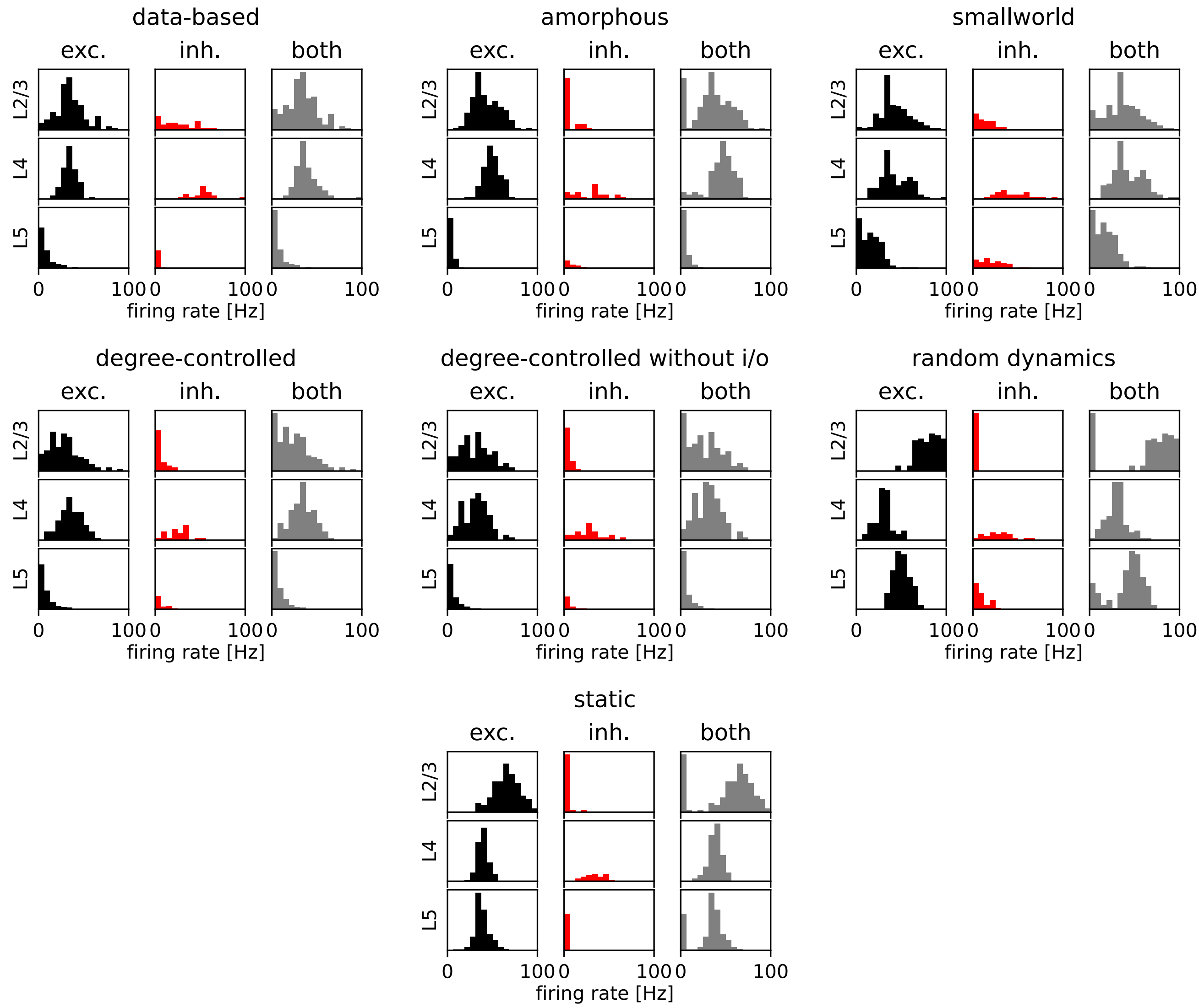

Figure S2. Firing rate histograms of the control circuits consisting of Hodgkin-Huxley neurons without conductance noise.

Tasks/circuits	Amorphous	Small-world	DC	DC (no io)	Random dynamics	Static synapses
memory	-25.8	-25.8	-7.9	-28.6	-46.7	-17.3
nonlinear	-30.1	-31.6	-22.8	-29.2	-52	-18.9
other	-5.1	-12.2	-4	-17	-39.7	-1.6
all	-16.2	-20	-8.9	-22.8	-44.2	-9.7

Table S1. Performance measures for all control networks consisting of Hodgkin-Huxley neurons without intrinsic conductance noise expressed as average difference (in percent) from the performance of the data-based circuit. All values are averaged over 20 runs.

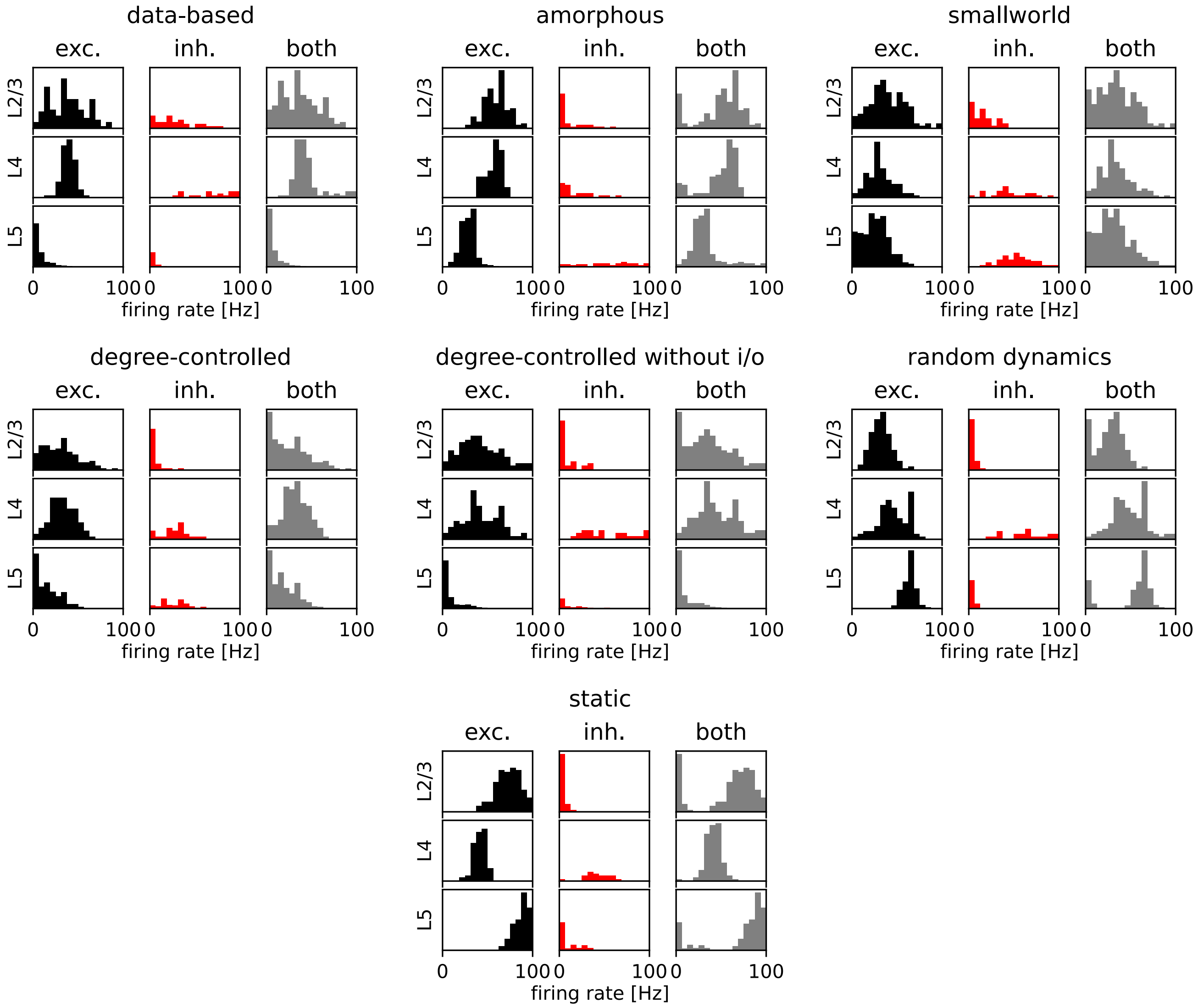

Figure S3. Firing rate histograms of the control circuits consisting of integrate-and-fire neurons.

Tasks/circuits	Amorphous	Small-world	DC	DC (no io)	Random dynamics	Static synapses
memory	-34.6	-23.6	-9.5	-31.3	-59.8	-24.8
nonlinear	-42.8	-21.8	-28.8	-28.8	-62.3	-27
other	-8.6	-9.9	-6.6	-18.1	-46.3	-8
all	-23.7	-16.6	-12	-24.5	-53.8	-17.2

Table S2. Performance measures for all control networks consisting of integrate-and-fire neurons expressed as average difference (in percent) from the performance of the data-based circuit. All values are averaged over 20 runs.
